# The Role of Hypoxia in Glioblastoma Radiotherapy Resistance

**DOI:** 10.3390/cancers13030542

**Published:** 2021-02-01

**Authors:** Agathe L. Chédeville, Patricia A. Madureira

**Affiliations:** 1 INSERM, UMR 1287, Gustave Roussy, CEDEX 94805 Villejuif, France; Agathe.CHEDEVILLE@gustaveroussy.fr; 2 Université Paris-Saclay, UMR 1287, Gustave Roussy, CEDEX 94805 Villejuif, France; 3 Gustave Roussy, UMR 1287, 114, Rue Edouard-Vaillant, CEDEX 94805 Villejuif, France; 4 Centre for Biomedical Research (CBMR), University of Algarve, Gambelas Campus, Building 8, Room 2.22, 9005-139 Faro, Portugal

**Keywords:** glioblastoma (GB), hypoxia, radiotherapy, Hypoxia Inducible Factor (HIF), radioresistance, glioma stem cells (GSC)

## Abstract

**Simple Summary:**

Glioblastoma (GB) is the deadliest type of primary brain tumor. Following diagnosis the patient´s median survival is only 16 months. There are currently around 450 clinical trials focused on the development of more effective therapies for GB. Nevertheless, radiotherapy remains the most clinically relevant and effective treatment for this devastating disease. Unfortunately, radiotherapy resistance (radioresistance) is frequently observed in GB patients. As a consequence tumor regrowth (recurrence) occurs and eventually the patient succumbs to the disease. It is crucial to fully understand the mechanisms by which GB cells become resistant to radiation in order to improve the sensitivity of these cells to radiotherapy and develop novel strategies to overcome this issue. In this review, we examined how low tumor oxygenation (known as hypoxia) which is a main feature of GB contributes to radioresistance to better understand the implications of this tumor microenvironment in GB treatment and recurrence.

**Abstract:**

Glioblastoma (GB) (grade IV astrocytoma) is the most malignant type of primary brain tumor with a 16 months median survival time following diagnosis. Despite increasing attention regarding the development of targeted therapies for GB that resulted in around 450 clinical trials currently undergoing, radiotherapy still remains the most clinically effective treatment for these patients. Nevertheless, radiotherapy resistance (radioresistance) is commonly observed in GB patients leading to tumor recurrence and eventually patient death. It is therefore essential to unravel the molecular mechanisms underpinning GB cell radioresistance in order to develop novel strategies and combinational therapies focused on enhancing tumor cell sensitivity to radiotherapy. In this review, we present a comprehensive examination of the current literature regarding the role of hypoxia (O_2_ partial pressure less than 10 mmHg), a main GB microenvironmental factor, in radioresistance with the ultimate goal of identifying potential molecular markers and therapeutic targets to overcome this issue in the future.

## 1. Introduction

Glioblastoma (GB) is classified by the World Health Organization (WHO) as a grade IV astrocytoma. It is the deadliest primary malignant brain tumor; the median patient survival time being only 16 months [1,2,3].

The current classification of the Central Nervous System (CNS) tumors by the WHO combines both their histopathological name followed by the characteristic genetic signature [4]. In accordance to this, GB is classified as GB, IDH-wildtype which is the most prevalent type, corresponding to approximately 90% of all cases, and GB, IDH-mutant. Over 90% of GB develop de novo, called primary GB. However, a minority of GB develop slowly from low-grade astrocytomas (secondary GB). Mutations in *IDH* are more frequently observed in secondary GB. There are three Isocitrate Dehydrogenase (IDH) enzymes (IDH1, IDH2 and IDH3), but only IDH1 and IDH2 enzymes have been shown to be mutated in GB. IDHs are responsible for the conversion of isocitrate to α-ketoglutarate. This process results in the production of the reducing agent Nicotinamide Adenine Dinucleotide Phosphate (NADPH). IDH-mutant enzymes have approximately 50% less activity compared to IDH wild-type (WT) proteins. This results in impaired production of bioenergy (NADPH) and intermediates and the production of the onco-metabolite 2-hydroxyglutarate (2-HG) which causes epigenetic changes. 2-HG via hyper-methylation leads to the loss of differentiation of GB cells. These changes caused by IDH mutation lead to reduced GB, IDH-mutant tumor growth compared to GB, IDH-WT. This consequently translates to a better overall prognosis for patients with GB, IDH-mutant [5].

GB has been further sub-classified into classical, proneural, neural and mesenchymal sub-types based on specific genetic signatures [6,7]. In this way, the classical sub-type is characterized by *Epidermal Growth Factor Receptor* (*EGFR*) gene amplification or mutation (leading to a constitutively active receptor) as well as by over-expression of neural stem cell genes. These include *Sonic hedgehog*, *Notch* and *NES* [7]. The proneural sub-type shows a gene signature characterized by over-expression of many proneural genes (e.g., *DCX*, *SOX, TCF4, ASCL1* and *DLL3*), amplification of the *Platelet-Derived Growth Factor Receptor A* (*PDGFRA*) gene and inactivation or loss of *TP53* [7,8]. In addition, IDH mutations are more common within the proneural subtype [9]. The expression of neuron gene markers, such as *NEFL*, *GABRA1*, *SYT1* and *SLC12A5* is a hallmark of the neural sub-type [7]. Lastly, the mesenchymal sub-type signature includes the expression of mesenchymal genes (e.g., *CHI3L1* and *MET*) [8] as well as inactivating mutations or deletion of the *Neurofibromin 1* (*NF1*) gene [7,10,11].

The current standard treatment for GB was established in 2005 by Roger Stupp and colleagues [12]. The so called “Stupp protocol” encompasses GB resection surgery (when possible as evaluated by MRI imaging) after what concurrent radiotherapy and chemotherapy with temozolomide (TMZ) are implemented. This is followed by additional 6 cycles of TMZ administration [12]. Radiotherapy alone can considerably increase patient survival. However, beneficial effects of chemotherapy with TMZ are most commonly observed in a sub-set of patients whose tumors contain *O6-methylguanine DNA methyltransferase* (*MGMT*) promoter methylation [1,9]. MGMT promoter methylation is also a prognostic factor associated with longer survival irrespective of TMZ treatment as well as longer post-progression survival (3–4 months) in patients with recurrent GB [13]. MGMT is a DNA repair enzyme that fixes damaged guanine nucleotides (O6-methylguanine) via transferring the methyl group at the O6 site of guanine to its cysteine residues. This reverts the gene mutation and subsequently avoids cell death induced by alkylating agents such as TMZ [14]. Several studies have demonstrated that regulation of *MGMT* expression in GB occurs mainly via epigenetic modification, namely through the methylation of CpG islands within the *MGMT* promoter. This leads to heterochromatinization which is accompanied by rearrangement and random localization of nucleosomes. Consequently, binding of transcription factors to the *MGMT* promoter becomes impaired [14].

The current standard radiotherapy dosage is a total of 60 Grays (Gy) in fractions of 2 Gy, administered 5 days a week for 6 weeks [12]. TMZ is concurrently administrated at a dose of 75 mg/m^2^ daily for 6 weeks. After a rest period of one month, TMZ chemotherapy is restarted at a dose of 150 mg/m^2^ daily for 5 days in the first month cycle. If this dose is tolerated, it can be increased up to 200 mg/m^2^ for 5 days per month. TMZ is administrated for 6 months after radiotherapy, but many physicians continue TMZ administration for 12–18 months even though it has not been proved to increase overall survival [12,15]. Worryingly, almost all GB patients develop resistance to current therapy and eventually succumb to the disease.

Despite the development of a large number of studies and hundreds of ongoing clinical trials, GB treatment has not changed since 2005 [9,12]. Understanding the complex biology of GB and the role of the tumor microenvironment is therefore crucial to develop novel and effective treatments in GB.

GB pathological features include hypoxic foci (where O_2_ partial pressure is less than 10 mmHg) containing necrotic cores. The hypoxic areas are surrounded by cell pseudopalisades and microvascular hyperplasia [9]. Research suggests that cellular pseudopalisades constitute invasive fronts of the tumor that likely originated from GB cells migrating away from the hypoxic regions. These cells over-secrete proangiogenic factors leading to an intensified form of angiogenesis which is known as microvascular hyperplasia [9].

The GB hypoxic microenvironment has been shown to be highly associated with tumor invasion and resistance to chemo- and radiotherapy which are the main causes of death in GB patients [16,17]. Importantly, dynamic contrast enhanced MRI analyses have indicated that Hypoxia Inducible Factor 1 (HIF-1) (hypoxia marker) and Vascular Endothelial Growth Factor A (VEGFA) staining and tumor vascularity significantly correlate with worse progression-free and overall GB patient survival [18,19].

In this review article, we examined the existing literature regarding the role of hypoxia in supporting radiotherapy resistance in GB with the aim to better understand the implications of this tumor microenvironment in GB treatment and recurrence.

## 2. Basic Principles of Cancer Radiotherapy

Radiotherapy is currently the major and most effective treatment modality for GB patients. Nevertheless, radioresistance remains a major clinical problem for these patients.

Ionizing radiation (IR) was discovered just before the turn of the 20th century by Marie and Pierre Curie and Wilhelm Conrad Roentgen [20]. It was during the 1920s that cancer radiotherapy was piloted and significantly evolved [20] due to several technologic and research advances. These included the invention by Coolidge et al. of a sealed-off vacuum x-ray tube which could be operated at 180,000 to 200,000 volts which introduced the kilovoltage era in radiotherapy. Another important advancement that contributed to the effectiveness of radiotherapy at that time was the development of the first quantitative methods for the measurement of radiation dose and of the first physical unit of dose, the roentgen (later replaced by the rad). Lastly, the experimental and clinical radiobiology research work led by Claude Regaud at the Fondation Curie in Paris pioneered the procedure and development of fractionated radiotherapy which is still currently in use [20].

The ability of IR to kill tumor cells relies mainly on its DNA damaging effects. This damage can occur either directly on the DNA molecules (accounting for 30–40% of lesions), or indirectly through the generation of free radicals such as reactive oxygen species (ROS) or reactive nitrogen species (RNS) that in turn damage the DNA molecules (responsible for 60–70% of lesions) [21,22].

Water radiolysis is a main process in the formation of free radicals (ROS) by IR, resulting in the formation of electrons, H˙ atoms, OH˙ radicals, H_3_O^+^, OH^−^ ions and dihydrogen (H_2_) and hydrogen peroxide (H_2_O_2_) molecules [23].

IR produces a spectrum of DNA base lesions, the most prevalent being 8-oxo-guanaine (8-oxoG), thymine glycol (5,6-dihydroxy-5,6-dihydrothymine) and formamidopyrimidines [4,6-diamino-5-formamidopyrimidine (FapyAde) and 2,6-diamino-4-hydroxy-5-formamidopyrimidine (FapyGua)] [24]. In addition, IR produces DNA Single Strand Breaks (SSBs) that have a unique signature, generating 3′ phosphate or 3′-phosphoglycolate ends rather than 3′-OH ends. Particularly important IR induced lesions are double strand breaks (DSBs) which occur due to multiple damaged sites closely located on both strands of the DNA molecule [24]. DSBs are more difficult to repair compared to SSBs leading to cancer cell death [25]. DSBs typically trigger DNA-damage responses (DDR). However, when DSBs cannot be efficiently repaired by the cellular DDR mechanisms, irradiated cells undergo the so-called mitotic catastrophe which is a major cell death mechanism caused by IR-induced DNA damage [26].

Typically, a dose of 1Gy of X-ray radiation produces around 3000 damaged bases, 1000 SSBs and 40 DSBs [22].

The decreased oxygen levels observed in hypoxic GB cells, lead to resistance to IR due to the reduced availability of oxygen which is needed to stabilize the DNA strand breaks caused by radiotherapy [27]. Under normoxic conditions (physiological or normal oxygen levels, where tissue oxygenation is around 40 mmHg), cells are vulnerable to IR due to oxygen fixation leading to irreversible DNA damage. However, under hypoxic conditions (where O_2_ partial pressure (pO_2_) is below 10 mmHg), there is diminished production of DNA radicals due to the low levels of oxygen and subsequently cells become more resistant to radiotherapy [28]. Consequently, the radiation dose required to achieve the same biological effect is about three times higher in the absence of oxygen as compared to physiological oxygen levels [27].

## 3. Hypoxia in GB

Tumor hypoxia is a hallmark of GB and mainly occurs due to the abnormal neovascularization observed within these tumors [29]. The blood vessels that feed the GB are typically highly permeable and easily collapsible due to the excessive recruitment and proliferation of endothelial cells (caused by excessive secretion of VEGFA by the tumor cells) and lack of pericyte coverage (which are cells that provide support to the blood vessels) [30,31,32]. In addition, these vessels exhibit larger diameters and possess thicker basement membranes when compared to physiological brain blood vessels [33]. As a consequence, the occurrence of microvascular thromboses and vessel occlusions are frequently observed in GB [34] which significantly impede blood flow leading to a heterogeneous microenvironment regarding tumor oxygenation [35]. Moreover, the anarchic organization and instability of the vascular system within the tumor can lead to dynamic phases of hypoxia and then reoxygenation within the different tumor fractions, known as “cycling hypoxia” [36].

### 3.1. Regulation of HIF Transcription Factors

**Figure 1:**
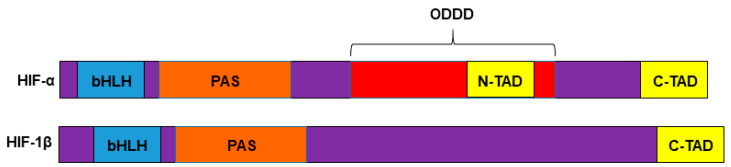
HIF protein domain structures. HIF-1α, HIF-2α, and HIF-1β subunits contain a bHLH domain (blue box), a PAS domain (orange box) and a C-TAD domain (yellow box). In addition, HIF-1α and HIF-2α subunits contain an ODDD (red box) and N-TAD (yellow box) domains. Different variants of the HIF-3α subunit containing diverse deletions of the domains shown in the figure have been shown to exist.

The cellular response to hypoxic stress is largely orchestrated by the HIF transcription factors. HIFs are heterodimers composed of an α subunit (e.g., HIF-1α, EPAS1/HIF-2α, or HIF-3α) which is negatively regulated by oxygen (O_2_) and a β subunit, HIF-1β, also known as aryl hydrocarbon receptor nuclear translocator (ARNT), which is expressed constitutively in cells [37,38]. Regarding protein domain structures, HIF-1α, HIF-2α, and HIF-1β subunits all have a Per-Arnt-Sim (PAS) and a basic Helix-Loop-Helix (bHLH) domains which are involved in the heterodimer assembly and binding to Hypoxia Responsive Elements (HRE) within HIF target gene promoters and a characteristic C-Terminal Domain (C-TAD) (Figure 1). Each HIF-α subunit also contain an Oxygen-Dependent Degradation Domain (ODDD) that is involved in the degradation of these proteins in the presence of normal levels of oxygen, and a specific N-Terminal Domain (N-TAD) (Figure 1). N-TAD and C-TAD domains are able to interact with p300/CBP HIF transcriptional coactivators [39,40,41]. Different variants of the HIF-3α subunit containing diverse deletions of the domains described above have been shown to exist (reviewed in [42]).

**Figure 2:**
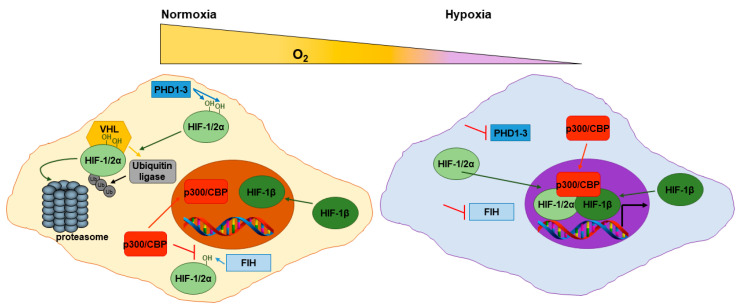
Regulation of HIF transcription factors. In normoxic cells, the PHD1-3 hydroxylate two prolyl residues within the HIF-α subunit enabling the binding of the VHL protein to the HIF-α subunit. VHL recruits E3 ubiquitin ligases that target HIF-α for degradation via the proteasome. In hypoxic cells, PHD1-3 activity (which is oxygen dependent) is inhibited. This leads to HIF-α stabilization and translocation to the nucleus where it binds to the HIF-1β subunit and p300/CBP cofactors. HIFs bind to HRE within their target gene promoters. In normoxic cells, FIH hydroxylates an asparagine residue within the HIF-α subunit. This blocks the interaction between HIF and the transcriptional activators, p300/CBP.

HIF transcriptional activity is highly dependent on the degradation (negative regulation) or stabilization (positive regulation) of the HIF-α subunit which is regulated by intracellular levels of oxygen. In normoxic cells, the Prolyl Hydroxylases 1-3 (PHD1-3) hydroxylate two prolyl residues within the HIF-α subunit. This enables the binding of the Von Hippel-Lindau (VHL) protein to the HIF-α subunit and the subsequent recruitment of E3 ubiquitin ligases that target HIF-α for degradation via the proteasome (Figure 2) [43,44,45]. Hypoxia inhibits PHD activity and consequently leads to HIF-α stabilization and translocation to the nucleus where it binds to the HIF-1β subunit and p300/CBP cofactors. HIFs bind to HRE within their target gene promoters orchestrating the hypoxic response [46,47,48]. The hydroxylase, Factor-Inhibiting HIF (FIH) has also been shown to regulate HIF activity in an oxygen dependent manner. In normoxic cells, FIH hydroxylates an asparagine residue within the HIF-α subunit which impairs the interaction between HIF and the transcriptional activators, p300/CBP subsequently negatively impacting on HIF transcriptional activity (Figure 2) [49,50].

HIF-1 and HIF-2 are considered the main regulators of the hypoxia response [37,38], while the existence of multiple variants of HIF-3α has highlighted that HIF-3 can function in some cases as a transcriptional activator whereas certain variants can act as dominant negative regulators of HIF-1 and/or HIF-2 transcriptional functions [42,51].

### 3.2. Hypoxia Independent HIF Activation in GB

**Figure 3:**
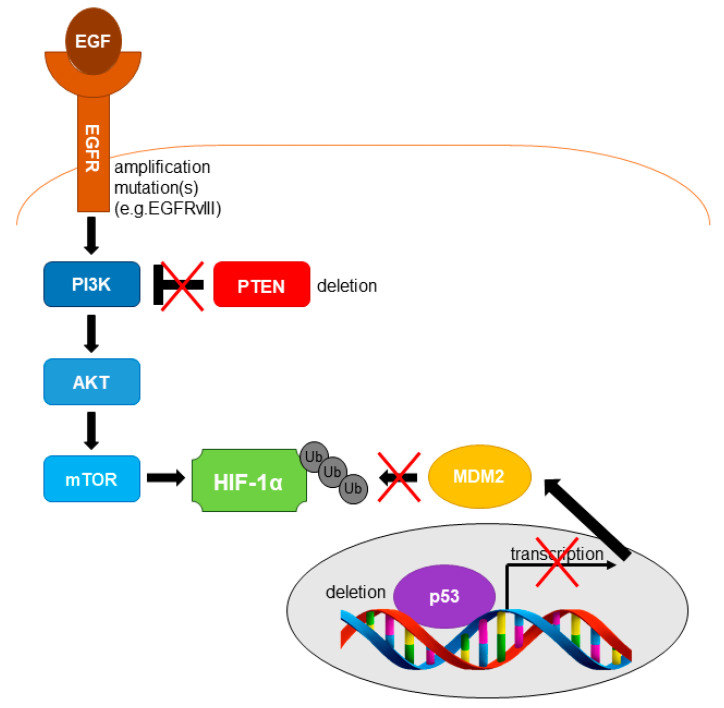
Genetic alterations leading to HIF activation in GB. Activation of the Epidermal Growth Factor Receptor (EGFR) by *EGFR* gene mutation (e.g., EGFRvIII) and/or amplification frequently occurs in GB cells resulting in activation of the PI3K/AKT/mTOR pathway with the subsequent accumulation of HIF-1α levels. The tumor suppressor Phosphatase and Tensin homolog (*PTEN*) gene is deleted in 20–40% of GBs. PTEN is the main inhibitor of the PI3K/AKT signaling pathway. Consequently, loss of *PTEN* will also lead to accumulation of HIF-1α via the PI3K/AKT/mTOR pathway. Loss of p53 inhibits MDM2-mediated ubiquitination of HIF-1α leading to its accumulation and increased HIF activity in GB cells.

Several genetic alterations leading to HIF activation, even in the absence of hypoxia, have been reported in GB (Figure 3). These include the activation of the EGFR (either by amplification or mutation of the *EGFR* gene) and the loss of the tumor suppressor genes *TP53* and Phosphatase and Tensin homolog (*PTEN*) [52,53,54]. The most common *EGFR* mutation observed in GB is the deletion of exons 2–7 (EGFRvIII) resulting in the expression of a constitutively active and ligand independent EGFRvIII receptor [55]. EGFR signaling leads to the up-regulation of HIF-1α levels via the activation of the PI3K/AKT/mTOR pathway [56,57]. Depletion of *PTEN* has been reported in about 20–40% of GB [53]. PTEN constitutes the main negative regulator of the PI3K/AKT signaling pathway. Therefore, loss of *PTEN* promotes the up-regulation of HIF-1α due to enhanced activity of the PI3K/AKT/mTOR pathway which is observed in the absence of PTEN protein. Loss of the *TP53* gene has been linked to HIF-1α stabilization due to down-regulation of *MDM2* transcription and subsequent inhibition of MDM2 mediated ubiquitination and degradation of HIF-1α (Figure 3) [54].

### 3.3. HIF Transcriptional Targets in GB

HIFs induce the transcription of hundreds of genes involved in the regulation of main cellular processes including angiogenesis, glycolysis, autophagy, motility and invasion, chemo- and radioresistance [58,59].

**Figure 4:**
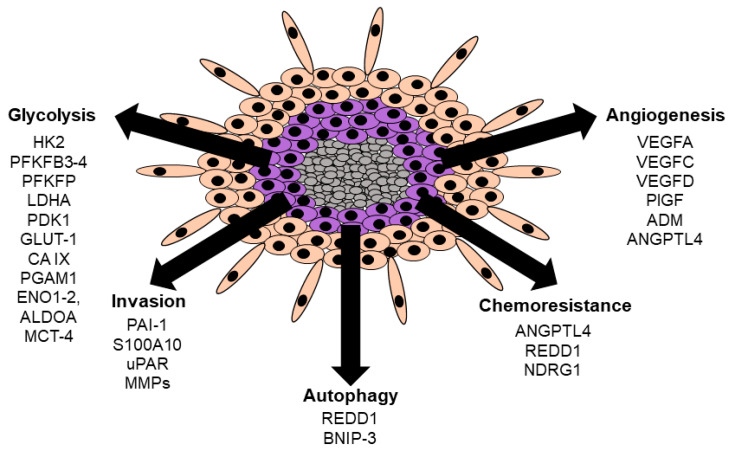
Hypoxia induced gene/protein expression in GB cells. Necrotic cells are represented in grey, hypoxic cells are represented in purple, and normoxic cells are represented in peach color.

Several studies using GB cell lines and/or clinical samples support a hypoxia triggered metabolic switch towards glycolysis including the up-regulation of HK2, PFKFB3, PFKFB4, PFKFP, LDHA, PDK1, SLC2A1/GLUT-1, CA9/CA IX, PGAM1, ENO1, ENO2, ALDOA and SLC16A3/ MCT-4 genes and proteins (Figure 4) [9,59,60]. Hypoxic up-regulation of many pro-angiogenic genes and proteins is also commonly observed in GB. These include VEGFA, VEGFC, VEGFD, PGF/PlGF, ADM and ANGPTL4 (Figure 4) [32,59,61,62]. GB is a highly invasive tumor and hypoxia has been shown to induce proteins of the plasminogen system. These include the plasminogen receptor, S100A10, the receptor for the urokinase Plasminogen Activator (uPA), uPAR and the Plasminogen Activator Inhibitor-1 (PAI-1) (Figure 4) [59,63,64]. The co-localization of S100A10 with uPAR at the outer cell membrane has been shown to promote the generation of the serine protease, plasmin by putting plasminogen (inactive form of plasmin) and its activator, uPA into close proximity. This leads to the subsequent degradation of the Extra-Cellular Matrix (ECM) by plasmin which is a critical step in GB cell invasion. Importantly, plasmin also has the capacity to cleave and activate many pro-MMPs, further accelerating ECM degradation [65,66]. The GB hypoxic environment has also been shown to promote the up-regulation of autophagy genes including *BNIP-3* and *DDIT4* (Figure 4) [59,67,68]. Several studies support that during hypoxic stress autophagy allows the recycling of cellular components which is critical for cell survival under oxygen and nutrients limiting conditions. Association of GB hypoxia with chemoresistance has also been demonstrated. Several reports have shown that hypoxic induction of ANGPTL4, DDIT4 and NDRG1 lead to resistance to chemotherapy (Figure 4) [59,69,70,71,72,73].

## 4. The Role of Hypoxia in GB Radioresistance

The molecular mechanisms by which GB becomes resistant to radiotherapy are still not fully understood. However, it has been shown that radioresistance can at least in part be due to the presence of hypoxic regions within the tumor [74].

Hypoxia contributes to radioresistance by controlling several cellular processes including regulation of the cell cycle, inhibition of apoptosis and senescence, regulation of autophagy and antioxidant/redox activity, promoting invasion and cancer cell stemness. In addition, radiotherapy is more efficient in rapidly proliferating cells as compared to slow-proliferating, quiescent and stem-like cells that are localized in the most hypoxic regions of the tumor [28].

### 4.1. The Role of Cell Cycle Regulation Proteins in Hypoxia Induced Radioresistance

**Figure 5:**
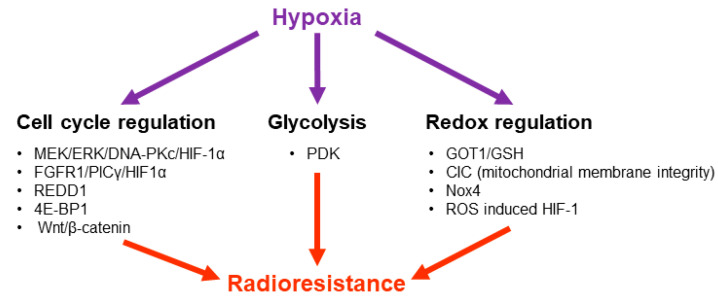
The role of hypoxia in GB radioresistance. Cell cycle regulation, glycolysis and Redox/ROS regulatory mechanisms have been shown to support hypoxia dependent radioresistance in GB cells.

**Figure 6:**
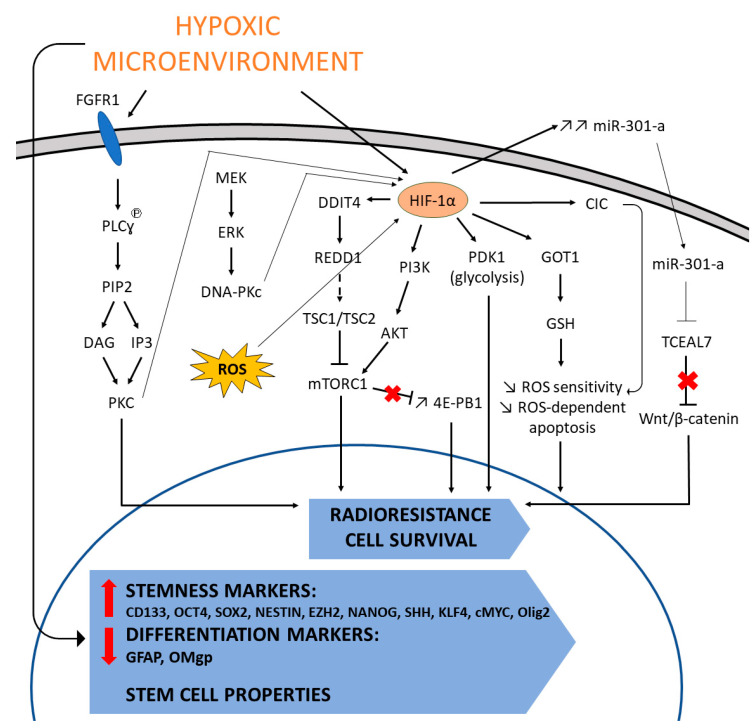
Hypoxia induced mechanisms leading to radioresistance in GB. The hypoxic environment induces several mechanisms involved in radioresistance including mechanisms involved in cell cycle regulation, Redox regulation, glycolysis and the maintenance of GSCs.

Several molecular mechanisms involved in cell cycle regulation have been shown to play a role in hypoxia induced radioresistance in GB (Figure 5 and Figure 6). A study showed that MEK/ERK inhibition either by treatment with the drug, U0126 or downregulation of ERK by siRNA significantly enhanced the radiosensitivity of hypoxic T98G, U87MG and U138MG GB cells [75]. Using a combination of siRNA approaches and chemical inhibitors these authors further mapped the MEK/ERK/DNA-PKc/HIF-1α functional interplay in hypoxia dependent GB radioresistance.

Another report highlighted the role of the Fibroblast Growth Factor Receptor 1 (FGFR1) in Phospholipase C Gamma (PlCγ)/HIF1α-dependent GB radioresistance [76]. FGFR signalling is involved in the regulation of cell proliferation, differentiation, migration, angiogenesis and tissue injury repair. PlCγ binds through its SH2 domain to a phosphotyrosine residue within the C-terminal tail of FGFRs and is phosphorylated at the tyrosine residues by the activated receptor tyrosine kinase. This phosphorylation activates PlCγ which then hydrolyzes phosphatidylinositol 4,5-bisphosphate (PIP2) to generate inositol1,4,5-trisphosphate (IP3) and 1,2-diacylglycerol (DAG) leading to Protein Kinase C (PKC) activation. Using in vitro and in vivo knockdown approaches, the authors were able to demonstrate that FGFR1/PlCγ/HIF1α signalling pathway confers radioresistance to GB cells (e.g., U87, LN18) and derived tumor mouse xenografts via controlling radiation-induced centrosome overduplication and radiation-induced mitotic cell death.

We recently observed a significant increase in *DDIT4* expression in hypoxic GB cells (e.g., U87, SEBTA-003, SEBTA-023, UP-007, UP-029) as compared to their normoxic counterpart control cells [59]. These data supported a previous report using U87 cells [67]. We also observed high expression of *DDIT4* in GB patient clinical samples, particularly in GB hypoxic core region as compared to normal brain specimens [59]. *DDIT4* gene product is the protein, REDD1 which is involved in the activation of the Tuberous Sclerosis 1/2 (TSC1/TSC2) complex, a main negative regulator of mTORC1 [77]. Even though over-expression of REDD1 in GB has been linked to chemo- and radioresistance the molecular mechanisms involved in these outcomes are still not fully understood [70,78].

Phosphorylation of 4E-BP1 occurs after activation of mTORC1, which functions downstream of the PI3K/AKT and AMPK kinase signaling pathways [79]. As previously mentioned, the PI3K/AKT signaling pathway is frequently activated in GB. Consequently, this leads to increased rates of cap-dependent translation in an mTORC1/4E-BP1–dependent manner. Using a mouse U87 GB xenograft model, a study has shown that 4E-BP1 promotes hypoxia dependent radioresistance [80]. These authors found that loss of 4E-BP1 expression by siRNA did not significantly affect in vitro growth of U87 GB cells, but significantly enhanced the growth of U87 tumor xenografts. Furthermore, 4E-BP1 knockdown U87 cells were significantly more sensitive to hypoxia-induced in vitro cell death. Most importantly, 4E-BP1 knockdown cells produced tumors with reduced fractions of radioresistant hypoxic cells.

The exosomal secretion of micro-RNA-301-a (miR-301-a) by hypoxic GB cells (e.g., U87, LN229, U251) has also been shown to lead to radioresistance [81]. Importantly, clinical analysis revealed higher levels of miR-301a expression in glioma samples with high HIF-1α levels and the percentage of serum exosomal miR-301a (low versus high) was distributed according to the HIF-1α immunohistochemistry score. In vitro studies demonstrated that miR-301a expression is regulated by HIF-1α. Furthermore, miR-301a directly repressed the promoter of the tumor suppressor gene, *TCEAL7* whose encoded protein binds to β-catenin inhibiting its translocation from the cytoplasm to the nucleus. Therefore, negatively regulating the Wnt/β-catenin signaling pathway. In summary, targeting of *TCEAL7* gene expression by miR-301a secreted by hypoxic GB cells induced enhanced activation of the Wnt/β-catenin signaling axis leading to radioresistance.

### 4.2. The Role of Glycolysis in Hypoxia Induced Radioresistance

As aforementioned, hypoxia induces a metabolic reprogramming towards glycolysis in GB cells. Interestingly, a research study has shown that modulation of the glucose metabolism can sensitize GB cells to IR (Figure 5 and Figure 6) [82]. Treatment of GB cells (e.g., U87, U251, LN229, DBTRG) with dichloroacetate, a PDK inhibitor, in combination with radiotherapy reversed the radiotherapy-induced glycolytic shift in these cells and inhibited their clonogenicity in vitro. Investigation into the molecular mechanism of action revealed that dichloroacetate sensitized GB cells to radiotherapy by inducing G2–M phase cell-cycle arrest, reducing mitochondrial reserve capacity, and increasing oxidative stress and DNA damage in these cells [82]. In vivo studies using a mouse xenograft model showed that radiotherapy in combination with dichloroacetate improved the survival of orthotopic GB-bearing mice [82]. Taking into account that hypoxia constitutes a major microenvironmental factor that triggers glycolysis in GB (including the specific up-regulation of PDK1 [59]) this report provides encouraging data regarding targeting the glycolytic metabolism in order to sensitize hypoxic GB cells to radiotherapy.

### 4.3. The Role of ROS Regulatory Systems in Hypoxia Induced Radioresistance

Several studies have shown that the regulation of intracellular ROS levels plays a key role in hypoxic GB radioresistance (Figure 5 and Figure 6). ROS are radical and non-radical oxygen-containing chemical molecules with different degrees of reactivity, including biologically relevant molecules such as superoxide anion (O_2_^−^), hydroxyl radical (∙OH) and hydrogen peroxide (H_2_O_2_) [58]. Of note, H_2_O_2_ constitutes a key second messenger in many cell signaling pathways [58,83]. However, due to their reactive properties ROS contribute to protein oxidation, lipid peroxidation and/or DNA damage that can ultimately result in either cell death or tumorigenesis (due to DNA mutagenesis) [58]. To overcome this issue, cells possess several antioxidant systems that inactivate ROS and recycle oxidized molecules (reviewed in [58]). A study showed that exposure of T98G GB cells to cycling hypoxia induced the up-regulation of the aspartate-aminotransferase Glutamic-Oxaloacetic Transaminase 1 (GOT1) protein, leading to increased levels of the antioxidant protein, glutathione (GSH), decreased intracellular ROS levels and enhanced radioresistance [84]. Most importantly, targeting glutamine-dependent antioxidant capacity or glutathione metabolism reversed the cycling hypoxia induced GB cells radioresistance. Exposure to either acute or cycling hypoxia was shown to trigger the up-regulation of the mitochondrial Citrate Carrier (CIC) and IDH2 in T98G GB cells in vitro [85]. CIC protein belongs to the large family of mitochondrial metabolite carriers. It is a mitochondrial transmembrane protein whose main function is to mediate the exchange of mitochondrial citrate for cytosolic malate. This process is accompanied with the transport of one proton and therefore can influence the mitochondrial membrane potential [86]. In fact, the tumorigenic activity of CIC has been shown to be linked to its role in mitochondrial membrane integrity [87] which is crucial to inhibit ROS induced apoptosis.

NADPH oxidase subunit 4 (Nox4) has been shown to mediate cycling (intermittent) hypoxia induced radioresistance in GB cells [88]. In this report, the GBM8401 and U251 cell lines were stably transfected with a dual hypoxia HIF-1 signaling reporter construct. The mouse tumor xenograft studies showed that Nox4 was highly expressed in the cycling hypoxic areas within the tumor microenvironment. In addition, when compared to the normoxic or acutely hypoxic GB cells, the cycling hypoxic GB cells derived from tumor xenografts showed significantly higher expression of Nox4, enhanced ROS levels and increased radioresistance which was reversed by Nox4 suppression in intracerebral GB bearing mice [88].

Another report revealed that hypoxia increased U87 GB cell radioresistance in vitro and in vivo via long-term induction of HIF-1 signaling transduction in a ROS dependent-manner [89]. These authors performed clonogenic survival assays to show that hypoxia pretreatment of U87 cells significantly increased GB cell resistance to IR compared with normoxic U87 control cells. To determine whether HIF-1 was a crucial mediator of hypoxia-induced radioresistance in U87 cells, they used a HIF-1 siRNA approach. Hypoxic HIF-1 knockdown U87 cells showed similar sensitivity to IR as compared to normoxic HIF-1 knockdown U87 control cells, indicating that the increased radioresistance observed in hypoxic U87 cells was mediated by HIF-1. The authors further confirmed these results in vivo using a mouse xenograft model [89].

### 4.4. The Role of Glioma Stem Cells in Radioresistance

As early as 1997, Bonnet and Dick described for the first-time leukemic cells that could transplant leukaemia in vivo into immunodeficient mice [90]. These cells were called “cancer stem cells” (CSCs) and were later identified in different types of cancer including breast, colon, lung, as well as in CNS cancers such as GB [91]. CSCs are slow-dividing small subpopulations of tumor cells that have the ability to undergo asymmetric cell division for self-renewal and multilineage differentiation giving rise to more mature cancer cells that constitute the bulk of the tumor [92,93]. The CSC hypothesis states that these cells have the ability to generate the cellular heterogeneity which is commonly observed within tumors. CSCs, called Glioma Stem Cells (GSCs) in GB, are suspected of being a main cause of tumor recurrence after treatment.

GSCs have been shown to have similar properties to Neural Stem Cells (NSCs). In addition to self-renewal capacity and multilineage differentiation potential, GSCs also express stemness markers involved in the regulation of specific signaling pathways, telomerase activity, expression of ABC transporter proteins, migration, secretion of cytokines, growth and pro-angiogenic factors [93].

It has been shown that hypoxia treatment of GB cells (e.g., SJ-1, U87) promoted CD133 expression (marker for GSC) and increased OCT4 and SOX2 mRNA levels, while promoting the loss of the glial differentiation marker, GFAP [94]. These data indicate that hypoxia promotes a GSC phenotype in GB. Another report took a broader approach by screening cell lines of different cancer types alongside human embryonic stem cells for overlapping changes of common genes when grown under hypoxic conditions [95]. The authors showed that OCT4, NANOG, SOX2, KLF4, cMYC, and miRNA-302 were all induced under hypoxic conditions in 11 different cancer cell lines from prostate, brain (U251 GB cell line), kidney, cervix, lung, colon, liver and breast tumors [95]. This report further supported a link between hypoxia and the stem cell phenotype by showing a correlation between the expression of HIFs and OCT4 [95].

Whether HIF-1α, HIF-2α or both transcription factors play a main role in hypoxia induced GSC phenotype is still a theme up for debate, with many studies showing differing results [29,95,96,97,98].

It has been well established that CSCs are significantly more radioresistant as compared to non-CSCs [26]. This is due to their enhanced DNA-repair capability, antioxidant defenses (in particular ROS scavenging systems) and self-renewal potential [99]. Interestingly, GSCs are found within a particular hypoxic environment, called a “niche”. This hypoxic niche forces the GSCs to develop mechanisms of survival and resistance to this harmful environment and also keeps the GSCs in a state of quiescence making them less vulnerable to the effects of radiotherapy [93]. Upon radiotherapy, the GSC populations containing advantageous genomic alterations that protect them against IR are selected and continue to sustain the tumor leading to recurrence.

Hypoxia has been shown to promote the undifferentiated state of GSCs through the activation of the Notch signaling pathway in a HIF dependent manner, contributing in this manner to GB radioresistance [100]. In summary, hypoxia is able of inducing the dedifferentiation of GB cells towards a GSCs phenotype, thus conferring aggressiveness and increased resistance to radiotherapy.

Interestingly, Dahan et al. demonstrated that IR itself is capable of inducing dedifferentiation of GB cells leading to overexpression of stem cell markers (SOX2, Nestin, SHH, Nanog, EZH2, Olig2) and a decrease in glial and neuronal differentiation markers (GFAP and OMgp, respectively) which resulted in increased tumorigenicity in vivo [101]. In addition, this team observed overexpression of survivin (a protein with anti-apoptotic properties that is expressed during neurogenesis) after irradiation of GB cells. Inhibition of this protein prevented the dedifferentiation of GB cells into GSCs [101]. This highlights a potential signaling pathway involved in the dedifferentiation of GB cells and a prospective therapeutic target which would make it possible to inhibit the acquisition of this stem-like phenotype highly linked to GB recurrence.

## 5. Conclusions

Targeting hypoxia-mediated radioresistance is considered an attractive approach to improve therapy outcome in GB. However, clinical trials evaluating the use of hypoxia-targeting agents have failed to reveal a benefit for GB patients, reviewed in [102]. This emphasizes the need for more effective and mechanism-based therapies to overcome hypoxia-induced radioresistance and the co-development of predictive biomarkers and improved imaging of heterogeneous GB hypoxia to guide radiotherapy protocols. As previously described, hypoxic areas of the tumor are more resistant to IR compared to normoxic areas. For this reason, increased radiation doses or number of radiation cycles within the GB hypoxic areas could be considered. Here we performed a review of the current literature, regarding the molecular mechanisms which are activated by hypoxia and that can potentially be targeted in the future to improve radiotherapy efficiency in GB patients. We revealed that several signaling pathways involved in cell cycle regulation were shown to provide radioresistance in hypoxic GB cells. These included the MEK/ERK/DNA-PKc/HIF-1α; PI3K/AKT/mTORC1/4E-BP1; and inhibition of *TCEAL7* transcription by miR-301-a leading to activation of the Wnt/β-catenin signaling pathway. The glycolytic metabolism which is triggered by hypoxia in GB cells was also shown to be involved in radioresistance and its inhibition showed promising in vivo results using a mouse xenograft model, providing radiosensitivity in the GB-bearing mice. These results are encouraging regarding the development of glycolytic inhibiting therapy approaches in combination with IR treatment. In addition, several ROS-dependent and Redox regulatory mechanisms were shown to play a role in hypoxic GB radioresistance. These included enhanced expression of GOT1 leading to increased levels of the antioxidant protein, GSH; up-regulation of CIC which has been linked to mitochondrial membrane integrity and inhibition of ROS-induced apoptosis; up-regulation of Nox4 and ROS-dependent up-regulation of HIF-1. Taken together these data highlight that targeting key Redox systems in combination with IR treatment might constitute a promising approach in GB therapy. Finally, the hypoxic microenvironment has been shown to play a role in GSC maintenance and quiescence which are associated with GB radioresistance. This occurs via inhibition of differentiation markers (e.g., GFAP, OMgp) and induction of stemness markers (e.g., CD133, OCT4, SOX2, NANOG, NESTIN, EZH2, SHH, KLF4, cMYC, Olig2).

In conclusion, targeting key cellular proteins/mechanisms involved in cell cycle regulation, glycolytic metabolism, Redox regulation and/or GSC maintenance in combination with IR treatment may potentially lead to the development of novel and effective therapies for GB patients.
